# Evaluation of microleakage in composite restorations with various adhesive systems: An *in vitro* comparison

**DOI:** 10.6026/973206300214035

**Published:** 2025-11-15

**Authors:** Rini Behera, Kunj Patel, Mohammed Khwaja Moinuddin, Sruthi Katamneni, Satabdi Pattanaik, Mrunal Dave

**Affiliations:** 1 Department of Conservative Dentistry and Endodontics, Institute of Dental Sciences, Siksha O Anusandhan (Deemed to be University), Bhubaneswar, Odisha, India; 2 Department of Public Health, University of Cincinnati, Cincinnati, Ohio, USA; 3 Department of Oral Health Sciences, Nizam's Institute of Medical Sciences, Hyderabad, Telangana, India; 4 Department of General Dentistry, Indiana University School of Dentistry, 4032 McDermott Rd Suite 200, Plano, TX 75024, USA; 5 Department of Conservative Dentistry and Endodontics, Institute of Dental Sciences, Khandagiri, Bhubaneswar, Odisha, India; 6 Department of Dentistry, Bethlehem Smile Design, Bethlehem, Pennsylvania, USA

**Keywords:** Composite restoration, microleakage, adhesive systems, universal adhesive, etch-and-rinse, self-etch, Class V cavity

## Abstract

Microleakage is a persistent challenge in adhesive dentistry and has a direct effect on the longevity of composite restorations. This *in vitro* study performed in accordance with the criteria of the preferred guidelines, investigated and compared, microleakage status of restorative bonded with etch-and-rinse, self-etch and universal adhesive systems. Twelve extracted human premolars were restored using standardized class V cavities and then utilized for dye penetration farming after thermocycling. Data analysis demonstrated statistically significant differences in sealing ability with respect to each adhesive group. Universal adhesives demonstrated a lower amount of microleakage than self-etch and etch-and-rinse systems. Microleakage is an important factor in adhesive bonding ultimately dictates failure or success the selection of an appropriate adhesive may relate to the reduction of adhesion and therefore increases the chance of restorative success.

## Background:

Microleakage is a main challenge to durability and clinical success of a resin composite restoration. It can cause sensitivity after operation, secondary caries and ultimately restorative failure [1]. Composite resins have achieved dominance in restorative dentistry because of their favorable esthetics, acceptable physical properties and ability to achieve a direct bond to tooth structure [2]. However, microleakage at the tooth-restoration interface continues to be a clinical problem for dental practitioners who can lead to subsequent caries, pulpal inflammation and/ or irritation, marginal enamel staining and loss of functionality [3]. The introduction of adhesive systems has greatly affected composite restoration success. Traditional three-step etch-and-rinse adhesives utilize phosphoric acid to condition enamel and dentin; etching is followed by rinsing, then bonding [4]. Three-step etch-and-rinse adhesives provide reliable bond strength but require multiple steps which rely on technique (and therefore may incur potential problems like postoperative pain/ discomfort and incomplete hybridization of the bonding) [5]. Self-etch adhesives were introduced to address the makeup of the technique-sensitive and this type of adhesive does not require rinsing of the phosphoric acid [6]. In one action self-etch adhesives remove the smear layer by demineralizing and allow resin infiltration into the layer. A self-etch type bonding agent could be considered less technique sensitive and can limit postoperative complications; however, the lower acidity of self-etching agents can negatively affect effective enamel bonding [7]. Recently developed universal adhesives allow for flexibility in use as etch-and-rinse, self-etch, or selective-etch systems. These products seek to preserve the benefits of prior generations of adhesive while removing clinical uncertainties but many questions remain about their long-term sealing ability and ability to reduce microleakage [8]. Many studies have compared the behaviour of adhesives among the different adhesive strategies, however variability in methodology, cavity restoration designs and evaluation methods have been found to create non-consistent results and continued debate [9]. Therefore, it is of interest to compare microleakage in composite restorations bonded with etch-and-rinse, self-etch and universal adhesive systems and determines the overall impact of these systems under standardized *in vitro* conditions.

## Materials and Methods:

Forty-five freshly extracted human premolars free from caries, cracks, or restorations were collected, cleaned and stored in distilled water until use. Standardized Class V cavities were prepared on the buccal surfaces of all teeth with dimensions of 3 mm mesiodistal width, 2 mm occlusogingival height and 2 mm depth using a diamond bur in a high-speed handpiece with water cooling to avoid heat generation. The specimens were then randomly allocated into three experimental groups, with 15 teeth in each group. Group I received the conventional etch-and-rinse adhesive system, where the cavities were etched with 37% phosphoric acid, rinsed, dried and adhesive applied according to the manufacturer's instructions. Group II was bonded with a self-etch adhesive system, applied directly without prior acid etching or rinsing. Group III utilized a universal adhesive system applied in the self-etch mode. In all groups, cavities were restored incrementally using a Nano hybrid composite resin of shade A2. Each increment was light-cured with an LED curing unit for 40 seconds to ensure adequate polymerization. Following placement, restorations were finished and polished using composite polishing discs. To simulate oral conditions, all samples were subjected to thermocycling for 500 cycles between 5°C and 55°C, with a dwell time of 30 seconds in each bath. After thermocycling, each specimen was coated with nail varnish except for a 1 mm area surrounding the restoration margins and immersed in 2% methylene blue dye solution for 24 hours. The teeth were then sectioned longitudinally through the center of the restorations and the degree of dye penetration at both occlusal and cervical margins was assessed under a stereomicroscope at 20x magnification. Microleakage was scored on a scale of 0 to 3, where 0 indicated no leakage and 3 indicated dye penetration extending up to the axial wall. Data obtained were analyzed using one-way ANOVA followed by Tukey's post hoc test with the significance level set at p < 0.05.

## Results:

The research studied microleakage in Class V cavities restored with three distinct adhesive systems, with significant group differences observed. Of the three systems assessed, universal adhesives consistently exhibited the least microleakage; self-etch showed a moderately moderate amount of microleakage and etch-and-rinse adhesives showed the highest microleakage values (p < 0.05). Universal adhesives performed consistently better than other adhesive systems at both occlusal and cervical margins. Self-etch adhesives showed a moderately lower microleakage when compared to etch-and-rinse, but etch-and-rinse adhesives showed the highest microleakage values at both occlusal and cervical margins. When compared to both occlusal and cervical margins, within each adhesive group, cervical margins demonstrated mildly higher microleakage, not statistically significant. Distributing 15 samples per group depended on comparative data. Overall, adhesive choice plays an important role in marginal sealing effectiveness. Universal adhesives behaved extremely well to minimize microleakage. Improved clinical outcomes with universal adhesive systems in Class V restorations may be possible, thus limiting marginal failure is dependent upon an understanding of adhesive strategy. The results showed significant differences in microleakage between the groups (p < 0.05). Universal adhesive demonstrated the least microleakage, followed by self-etch, whereas etch-and-rinse adhesives showed the highest leakage values.

Table 1 (see PDF) shows the distribution of the study samples into three groups based on the adhesive system used. Each group consisted of 15 standardized Class V cavities, with Group I bonded using the etch-and-rinse adhesive system, Group II with the self-etch adhesive system and Group III with the universal adhesive system. The uniform distribution ensured equal sample size which allowed for larger sample size sufficient enough to make a comparison of microleakage performance among adhesive strategies. Table 2 (see PDF) shows the mean microleakage scores (± SD) observed across the groups for each adhesive system. Group I (etch-and-rinse) had the mean microleakage (1.93 ± 0.64), followed by Group II (self-etch) with 1.41 ± 0.59. While Group III (universal adhesive) had mean microleakage score of 0.87 ± 0.52. The statistical analysis identified a significant difference of microleakage among the groups, with Group II as having significantly lower leakage in comparison to Group I (p = 0.04) and Group III had the best outcome with highly significant difference from Group I (p = 0.001). Table 3 (see PDF) presents the mean microleakage scores (± SD) for the three adhesive groups. Group I (etch-and-rinse) had the highest mean microleakage (2.14 ± 0.72) and Group II (self-etch) had a mean microleakage of 1.60 ± 0.61. Group III (universal adhesive) demonstrated the lowest mean microleakage (0.95 ± 0.48). Statistical comparison revealed a significant reduction in microleakage in Group II compared to Group I (p = 0.03), while Group III showed a highly significant reduction compared to Group I (p = 0.001), indicating its superior sealing ability. Table 4 (see PDF) compares the mean microleakage scores (± SD) between occlusal and cervical margins across the three adhesive groups. In Group I (etch-and-rinse), the cervical mean (2.14 ± 0.72) was slightly higher than the occlusal mean (1.93 ± 0.64), while Group II (self-etch) also showed a higher cervical mean (1.60 ± 0.61) compared to the occlusal mean (1.41 ± 0.59). Similarly, in Group III (universal adhesive), cervical scores (0.95 ± 0.48) were marginally greater than occlusal scores (0.87 ± 0.52). However, none of these differences reached statistical significance (p > 0.05), indicating that the adhesive systems performed comparably at both margins.

## Discussion:

In this investigation, microleakage rates of three adhesive niches were tested and compared for the standardized Class V restorations. The results indicated that the universal adhesive systems had microleakage scores that were substantially lower than either self-etch and etch-and-rinse systems, highlighting the imitating characterization of universal adhesives in both enamel and dentin modality contexts [10]. The etch-and-rinse adhesives had the greatest amount of leakage and demonstrated the greatest amount of leakage at the cervical margins, which also aligns with that of previous studies and the challenges of dentin bonding [11]. Self-etch systems had relatively better scores than etch-and-rinse; however, this could be due to a reduced potential to etch enamel, yet better dentin bonding due to porous demineralized dentin being infiltrated at the same time [12]. The universal adhesives had the best possible due to functional monomers such as 10-MDP that can chemically bond to hydroxyapatite and increase longevity of bonding [13]. It should also be noted that all cervical margins yielded consistently higher leakage values than occlusal margins and that all groups had cervical margins; this is an important observation and it affirms previous studies that recognized the challenges associated with sealing dentin and cementum-rich areas. Though this study was conducted *in vitro*, it gives us meaningful insights. While this investigation was undertaken *in vitro*, it contributes useful information for adhesive consideration. However, clinical extrapolation should be taken with caution because factors in the oral environment such as occlusal stresses, saliva and bacterial challenge may have implications on clinical outcomes.

## Conclusion:

Within the limitations of this *in vitro* study, universal adhesives showed the least microleakage compared to self-etch and etch-and-rinse systems. Adhesive choice plays a crucial role in the marginal integrity of composite restorations and can significantly affect their clinical longevity.
